# Prevalence and Predisposing Factors of *Helicobacter pylori* Infection in Symptomatic Patients: A Multicenter Study in Ghana's Upper East Region

**DOI:** 10.1002/puh2.70248

**Published:** 2026-04-27

**Authors:** Elvis Ramsy Awuni, Maxwell Akonde, Benedict Boateng Antuamwine

**Affiliations:** ^1^ Department of Biomedical Laboratory Sciences, School of Allied Health Sciences University for Development Studies Tamale Ghana; ^2^ Arnold School of Public Health, Epidemiology and Biostatistics University of South Carolina Columbia South Carolina USA

**Keywords:** Helicobacter pylori, infection, northern Ghana, predisposing factors, prevalence

## Abstract

**Background:**

*Helicobacter pylori* (*H. pylori*) infection is a major cause of dyspeptic disorders and remains highly prevalent in Ghana. However, data on its epidemiology in northern Ghana are limited. The aim of the study was to determine the prevalence of *H. pylori* infection and identify associated predisposing factors among dyspeptic patients in the Upper East Region of northern Ghana.

**Methods:**

A multicenter cross‐sectional study was conducted among 325 dyspeptic patients tested for *H. pylori* infection. Sociodemographic and clinical data were collected using a structured questionnaire, whereas stool samples were analyzed for *H. pylori* antigens using an immunoassay. Bivariate and multivariable logistic regression analyses were performed to identify factors associated with *H. pylori* infection.

**Results:**

The overall prevalence of *H. pylori* infection was 65.8% (214/325), with the highest prevalence observed in patients within the age category 1–20 years (40.7%). Major predisposing factors included use of public toilets (57.0%), consumption of untreated water (66.4%), and rural residence (56.5%). Consumption of food from street vendors was associated with a 2.27‐fold increased likelihood of infection (odds ratio [OR] = 2.27), whereas intake of fresh fruits and vegetables increased the risk by 4.81‐fold (OR = 4.81). Patients from larger households (≥13 members) were at greater risk of infection compared with those from smaller households (1–6 members) (OR = 0.31).

**Conclusion:**

The study reveals a high prevalence of *H. pylori* infection in Ghana's Upper East Region, which is strongly associated with poor sanitation and inadequate personal and food hygiene. Public health interventions aimed at improving sanitation, water quality, food safety, and living conditions are crucial for reducing transmission.

## Graphical abstract

A high *Helicobacter pylori* prevalence of 65.8% was detected among dyspeptic patients in northern Ghana, linked to poor sanitation, use of public toilets, consumption of street‐vended food, and drinking untreated water. These findings underscore the need for targeted public‐health interventions.

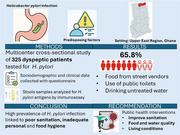

## 1 Background

*Helicobacter pylori* (*H. pylori*) is a ubiquitous and fastidious microaerophilic bacterium, that is, recognized as the most prevalent chronic bacterial pathogen in humans [1]. It is found mainly in the gastric antrum, and a major cause of gastritis, non‐ulcer dyspepsia, gastroesophageal reflux disease, and peptic ulcer [2]. Chronic infection with the organism can also result in gastric adenocarcinoma as well as mucosa‐associated lymphoid tissue, gastric lymphoma [3]. It has been reported that about two‐thirds of gastric cancer globally are caused by *H. pylori* [4]. The production of colonizing and virulence factors by the pathogen gives it an advantage to adhere, invade, and evade host defenses and cause tissue damage [5].

*H. pylori* infection has a global presence, but it is most common in developing countries with prevalence rates of up to 90% been recorded [6, 7]. However, prevalence of the infection varies between and within nations in relation to age, race, gender, ethnicity, and socioeconomic status [8]. Low socioeconomic status, overcrowded living conditions, poor hygiene, and insanitary conditions are among the factors favoring the spread of the infection [9]. Infections are usually acquired in early childhood in most countries [8]. However, the exact mechanism of transmission is unclear but the spread of the bacteria is mostly attributed to fecal–oral and oral–oral routes [10].

*H. pylori* colonization can result in acute gastritis, which normally presents as dyspepsia, heartburns, and nausea [11]. Dyspeptic symptoms secondary to *H. pylori*‐associated peptic ulcer have also been recorded [2]. Recurrent abdominal symptoms (non‐ulcer dyspepsia) also occur in patients infected with the organism [5]. Interestingly, prevalence of the infection among dyspeptic patients is on the high, especially in rural settings and among low socioeconomic groups [12]. Epidemiological reports on *H. pylori* infection in Africa, especially among symptomatic (dyspeptic) patients in Nigeria [12] and other African countries [5], have all shown high prevalence of the pathogen. The case in Ghana is not any different as reported by Darko et al. [13]. However, to the best of our knowledge and search, studies on the prevalence of *H. pylori* in Ghana are limited to the southern part of Ghana, whose socioeconomic, hygienic, and living conditions are different from the northern part of Ghana [1, 13, 14, 15].

There are disproportionately higher poverty rates in the northern parts of Ghana compared to the national average, with per capita income and household consumption expenditure among the lowest in the country [16]. The south enjoys relatively better access to improved water sources and sanitation facilities due to more extensive infrastructural investments. Urban areas such as Accra, Kumasi, and Takoradi particularly benefit from higher coverage of piped water, household latrines, and waste‐management systems [17]. In contrast, many communities in northern Ghana face inadequate water supply, seasonal shortages, and limited sanitation facilities. Open defecation is more prevalent in the northern regions, reflecting both infrastructural gaps and entrenched socioeconomic constraints. Poor waste management, limited drainage systems, and lower public‐health budgets in these regions increase exposure to environmental contaminants and sanitation‐related diseases, reinforcing regional disparities in health outcomes [18].

It is against this background that this study was aimed to evaluate the prevalence of *H. pylori* infection and associated predisposing factors among symptomatic patients in the Upper East region, one of the six regions in the northern part of Ghana. The outcome of this study will inform public health policy on preventive strategies targeted at *H. pylori* infection in the northern part of Ghana.

## 2 Methods

### 2.1 Study Design and Population

This was a cross‐sectional multicenter study conducted from March to June 2019 in patients who were at least 1 year old. The patients were recruited from three selected private major medical diagnostic facilities (Iqurate Medical Laboratories, Biomedilab Diagnostic Services, and Bioanalytic Diagnostic Services) within the Bolgatanga municipality of the Upper East region. These facilities provide diagnostic services across a diverse, cosmopolitan municipality characterized by a mix of ethnic, cultural, religious, and socioeconomic backgrounds. They also serve referral patients from the districts within the Upper East region, as well as parts of the Upper West and Northeast regions of northern Ghana. Participants were recruited from patients presenting with gastrointestinal symptoms who were referred for *H. pylori* testing. Asymptomatic patients and symptomatic patients who had used antibiotics, pain medications, nonsteroidal anti‐inflammatory drugs, bisphosphonates, or proton pump inhibitors in the past 2 weeks were excluded from the study.

### 2.2 Ethical Considerations

Ethical approval for the study was obtained from the Quality Assurance and Ethics Committee of the University for Development Studies, Tamale (SAHS‐QAERC‐MLS/600‐02/0015/2018‐2019). Administrative permission was obtained from each of the three facilities, whereas informed consent was obtained from each patient after explaining the objectives, benefits, risks, procedures, and confidentiality issues of the study to them. For respondents below 18 years, their parents or legal guardian consented on their behalf.

### 2.3 Sample Method and Size

Patients were recruited into the study employing a simple random sampling approach. A list of eligible patients was compiled from their referral records, and participants were randomly selected using a computer‐generated random number sequence to ensure unbiased selection. A sample size of 325 participants was determined based on an estimated prevalence of 75.4% [19] at 95% confidence interval (CI) and a 5% margin of error. To minimize the effects of non‐responsiveness, 14% of the calculated sample size was added.

### 2.4 Stool Specimen Collection and Laboratory Analysis

Participants were instructed to provide a fresh stool sample in a sterile, transparent, leak‐proof, and screw‐capped container. They were guided on how to collect the specimen aseptically and transport it promptly to the designated health facility, ensuring it remained uncontaminated and intact. Samples that were dried, contaminated, or submitted in inappropriate containers were excluded. All valid specimens were analyzed within 2 h of collection for *H. pylori* infection using the RapiCard InstaTest antigen detection kit (Diagnostic Automation Inc., USA), following the manufacturer's protocol.

### 2.5 Data Collection With Questionnaire

Sociodemographic and clinical data were obtained using a structured questionnaire administered to all participants. The questionnaire captured information on age (grouped as 1–20, 21–40, 41–60, and >60 years), gender (male/female), marital status (married/single), religion (Christian, Muslim, and Traditionalist), and occupation (farmer, trader, professional, unemployed, and retired). Household size was categorized into 1–6, 7–13, and >13 members. Environmental and lifestyle variables included residential area, source of drinking water (treated/untreated), and type of toilet facility (private, public, and open defecation). Information on dietary practices focused on whether participants consumed street foods, fresh fruits, or vegetables within the past 2 weeks. Clinical data covered medication use, gastrointestinal symptoms (heartburn, nausea, and vomiting), and *H. pylori* status, all documented as binary responses.

### 2.6 Statistical Analysis

Bivariate analysis was conducted to assess the association between participants’ sociodemographic characteristics and *H. pylori* status using the chi‐square (*χ* ^2^) test. The relationship between *H. pylori* status and the potential risk factors was further evaluated using logistic regression. Multivariable logistic regression with backward stepwise selection was applied, retaining variables with *p* ≤ 0.20. The final model included religion, occupation, household size, residential area, toilet facility type, water source, and consumption of street food and fresh fruits/vegetables. Crude and adjusted odds ratios (ORs) with 95% CIs were reported, with statistical significance set at *p* < 0.05. Analyses were performed using SPSS version 23 (IBM Corp., Armonk, NY, USA).

## 3 Results

A total of 325 dyspeptic patients with *H. pylori* test requisitions were enrolled, where we evaluated their sociodemographic and environmental characteristics in relation to *H. pylori* infection status. Overall, the study population was predominantly young to middle‐aged adults, with a slightly higher proportion of females (Table 1). Environmental risk factors, such as reliance on public toilet facilities, open defecation, and the use of untreated drinking water, were common among participants. The prevalence of *H. pylori* infection in the study population was 65.8%, with more frequent infections among individuals aged from 1 to 40 years old (78.7%), those residing in rural areas (56.5%), those using water from untreated sources (66.4%), and those who were unemployed (48.6%) as shown in Table 1. Interestingly, *H. pylori* infection status showed significant associations with residential area, household size, type of toilet facility, and source of drinking water (Table 1).

**TABLE 1:** Sociodemographic characteristics of the study participants stratified by *Helicobacter pylori* infection status.

Variable	Total (*n* = 325)	Positive (*n* = 214)	Negative (*n* = 111)	*χ* ^2^	*p* value
Age (years)					
1–20	123 (37.8)	87 (40.7)	36 (32.4)	5.502	0.139
21–40	133 (40.9)	80 (37.4)	53 (47.7)		
41–60	57 (17.5)	41 (19.2)	16 (14.4)		
>60	12 (3.7)	6 (2.8)	6 (5.4)		
Sex					
Male	146 (44.9)	94 (43.9)	52 (46.8)	0.252	0.616
Female	179 (55.1)	120 (56.1)	59 (53.2)		
Marital status					
Married	174 (53.5)	109 (50.9)	65 (58.6)	1.708	0.191
Single	151 (46.5)	105 (49.1)	46 (41.4)		
Religion					
Christian	152 (46.8)	97 (45.3)	55 (49.5)	1.988	0.370
Muslim	146 (44.9)	96 (44.9)	50 (45.0)		
Traditionalist	27 (8.3)	21 (9.8)	6 (5.4)		
Occupation					
Farmer/Rearing	60 (18.5)	42 (19.6)	18 (16.2)	5.376	0.251
Trader	64 (19.7)	41 (19.2)	23 (20.7)		
Unemployed	151 (46.5)	104 (48.6)	47 (42.3)		
Professional	40 (12.3)	23 (10.7)	17 (15.3)		
Retired	10 (3.1)	4 (1.9)	6 (5.4)		
Residential area					
Urban	106 (32.6)	59 (27.6)	47 (42.3)	7.479	0.024
Rural	169 (52.0)	121 (56.5)	48 (43.2)		
Zongo/Inner‐city^a^	50 (15.4)	34 (15.9)	16 (14.4)		
Household size					
1–6	116 (35.7)	62 (29.0)	54 (48.6)	13.677	0.001
7–13	152 (46.8)	107 (50.0)	45 (40.5)		
>13	57 (17.5)	45 (21.0)	12 (10.8)		
Toilet facility					
Private	78 (24.0)	41 (19.2)	37 (33.3)	8.772	0.012
Public	170 (52.3)	122 (57.0)	48 (43.2)		
Open defecation	77 (23.7)	51 (23.8)	26 (23.4)		
Water source					
Treated/Mechanized well	130 (40.0)	72 (33.6)	58 (52.3)	10.544	0.001
Untreated	195 (60.0)	142 (66.4)	53 (47.7)		

*Note:* Categorical data are presented as frequencies with percentages in parenthesis. *χ* ^2^ = chi‐square statistic.

^a^ Zongo/Inner city denotes living in congested, socioeconomically disadvantaged urban communities with poor housing and sanitation infrastructure.

The study also assessed dietary and clinical characteristics of the participants in relation to *H. pylori* infection. Consumption of street‐vended food and fresh fruits or vegetables was widely reported among participants. Gastrointestinal symptoms, such as heartburn, nausea, and vomiting, were common among the study participants but did not differ significantly by infection status. However, *H. pylori*‐positive individuals were more likely to report recent intake of street‐vended foods and fresh produce, with both dietary factors showing significant associations with infection as shown in Table 2.

**TABLE 2:** Dietary and clinical symptoms of study participants stratified by *Helicobacter pylori* infection status.

Variable	Total (*n* = 325)	Positive (*n* = 214)	Negative (*n* = 111)	*χ* ^2^	*p* value
Consume food from street vendors					
Yes	293 (90.2)	198 (92.5)	95 (85.6)	3.963	0.047
No	32 (9.8)	16 (7.5)	16 (14.4)		
Consume fresh fruits and vegetables					
Yes	285 (87.7)	201 (93.9)	84 (75.7)	22.554	<0.001
No	40 (12.3)	13 (6.1)	27 (24.3)		
Symptoms (heartburns, nausea, and vomiting)					
Yes	213 (65.5)	140 (65.4)	73 (65.8)	0.004	0.950
No	112 (34.5)	74 (34.6)	38 (34.2)		

*Note:* Categorical data are presented as frequencies with percentages in parenthesis. *χ* ^2^; chi‐square statistic.

Multivariable logistic regression further identified key predictors of *H. pylori* infection (Table 3). Dietary exposures remained significant among participants, with consumption of street‐vended food and fresh fruits or vegetables independently increasing the likelihood of infection. In contrast, smaller household size was associated with reduced odds of *H. pylori* positivity, suggesting a role of household crowding in transmission dynamics. These findings underscore the important role of dietary practices and household factors in shaping *H. pylori* infection risk within the study population.

**TABLE 3:** Multivariable logistic regression of possible predisposing factors of *Helicobacter pylori* infection.

Variable	COR (95%CI)	AOR (95%CI)
Religion		
Christian	0.50 (0.19–1.32)	0.51 (0.17–1.56)
Muslim	0.55 (0.21–1.45)	0.46 (0.15–1.41)
Traditionalist (reference)	1	1
Occupation		
Farmer/Rearing	3.50 (0.88–13.918)	3.44 (0.75–15.73)
Trader	2.67 (0.68–10.46)	2.86 (0.63–12.89)
Unemployed	3.32 (0.89–12.32)	3.80 (0.90–16.00)
Professional	2.03 (0.50–8.33)	2.26 (0.48–10.66)
Retired (reference)	1	1
Household size		
1–6	0.31 (0.15–0.64)	0.31 (0.14–0.70)^*^
7–13	0.63 (0.31–1.31)	0.59 (0.26–1.31)
>13 (reference)	1	1
Residential area		
Urban	0.59 (0.29–1.20)	0.98 (0.43–2.23)
Rural	1.19 (0.60–2.35)	1.33 (0.62–2.87)
Zongo/Inner‐city (reference)	1	1
Toilet facility		
Private	0.57 (0.30–1.08)	1.12 (0.49–2.59)
Public	1.30 (0.73–2.31)	1.31 (0.69–2.49)
Open defecation (reference)	1	1
Source of drinking water		
Treated/Mechanized well	0.46 (0.29–0.74)	0.60 (0.34–1.07)
Untreated (reference)	1	1
Consume food from street vendors		
Yes	2.08 (1–4.35)	2.27 (1.01–5.07)^*^
No (reference)	1	1
Consume fresh fruits and vegetables		
Yes	4.97 (2.45–10.10)	4.81 (2.21–10.50)^*^
No (reference)	1	1

Abbreviations: AOR, adjusted odds ratio; CI, confidence interval; COR, crude odds ratio.

* *p* value < 0.05.

Overall, these results highlight the combined influence of sociodemographic, economic, environmental, and dietary factors on *H. pylori* infection in the population, providing the basis for exploring potential transmission pathways and public‐health implications.

## 4 Discussion

*H. pylori* infection remains a major public health issue worldwide, particularly in low‐ and middle‐income countries where crowded living conditions and suboptimal sanitation facilitate transmission [20]. In our study of dyspeptic patients in northern Ghana, we observed a high infection prevalence of 65.8% and found significant associations with living conditions, particularly household size and dietary exposures, including consumption of street‐vended foods, fresh fruits, and vegetables.

Our study, arguably the first conducted in the northern part of Ghana, shows a higher prevalence of *H. pylori* infection among dyspeptic patients compared to similar studies from the southern regions of the country. Specifically, prevalence rates of 45.2% [13] and 51.3% [21] have been reported in Accra, the capital city, whereas a rate of 58.7% was documented in Kumasi [14]. The persistently higher prevalence of *H. pylori* infection in the Upper East region may be linked to several socioeconomic and environmental challenges, including poverty, poor sanitation and housing, overcrowding, and increased exposure to contaminated food and water.

Despite the high prevalence of *H. pylori* infection observed in this study, the likelihood of progression to gastric cancer may be relatively low, based on several reports. According to the Kumasi Cancer Registry (2015), gastric cancer represented only 3.2% of all cancer cases in the registry population [22]. Similarly, among dyspeptic patients referred for endoscopy in a tertiary hospital in Kumasi, southern Ghana, gastric cancer was diagnosed in 2.3% of cases, with no significant association observed between *H. pylori* infection and malignancy [14]. A similar pattern may be present in northern Ghana as well, where a study among patients undergoing upper gastrointestinal endoscopy reported a gastric cancer incidence of 3.6%, with 54.7% testing positive for Campylobacter‐like organisms based on histopathological assessment [23]. Taken together, these findings suggest that although *H. pylori* infection is highly prevalent, its contribution to gastric cancer in Ghana may be relatively modest.

Living standards are estimated to be lower in the northern parts of Ghana compared to the south [24]. This may compel individuals to rent substandard housing, leading to higher occupancy rates within a single dwelling. It was therefore not surprising that participants with smaller household sizes (1–6 members) were significantly less likely to test positive for *H. pylori* compared to those with larger household sizes (>13 members), reflecting 69% lower odds of infection. This trend suggests that crowded living conditions may increase the risk of *H. pylori* transmission through close interpersonal contact and shared household resources. These findings are consistent with previous studies suggesting that household crowding is a key predisposing factor for *H. pylori* infection [25].

Furthermore, individuals residing in severely deprived areas with inadequate sanitation are at a heightened risk of continuous exposure to contaminated food and water. For example, food sold by street vendors is more likely to be prepared or stored under unhygienic conditions. In the present study, participants who consumed street‐vended food were 2.27 times more likely to be infected with *H. pylori* compared to those who did not. Interestingly and notably, 92.5% of the infected participants reported consuming such foods. This finding was consistent with other studies that have associated increased consumption of street‐vended food with higher prevalence of *H. pylori* infection [26, 27]. These studies collectively demonstrate that street‐vended foods are more prone to contamination, highlighting their role as a major route of *H. pylori* transmission. In many low‐resource settings, particularly densely populated urban areas, street food vendors often operate without access to clean water, proper waste disposal, or formal food safety training. As a result, contaminated food can become a critical vector for pathogens, including *H. pylori*.

The present study also sheds light on the consumption of fresh fruits and vegetables as a significant risk factor for *H. pylori* infection. Because these foods are often eaten raw, inadequate washing or treatment can allow pathogens to remain on their surfaces. In densely populated communities with poor sanitation, individuals may be unable to follow recommended hygiene practices such as washing fruits and vegetables under running tap water due to limited access to potable water. Several studies in developing countries have similarly reported that consumption of raw vegetables is a high‐risk factor for *H. pylori* infection, with the risk increasing alongside the quantity of vegetables consumed [28, 29, 30]. These challenges are further compounded by socioeconomic constraints, including poverty, limited public health infrastructure, and weak regulatory enforcement, which perpetuate a cycle of foodborne illnesses among vulnerable populations. Addressing these issues requires a multifaceted approach involving community education, improved sanitation infrastructure, and strengthened policy implementation to promote safer food environments.

Whilst the study provides important public health findings, these findings should be interpreted considering several notable limitations. First, the study employed a cross‐sectional design and therefore is subject to limitations typical of this type of design, including recall bias and lack of temporality. Second, the stool antigen test method for *H. pylori* diagnosis rather than the gold standard urea breath test, which is recognized to provide more reliable and accurate results than many other tests, including the immunoassay. Finally, the study was conducted among symptomatic patients seeking healthcare, and thus its findings cannot be generalized to the entire population of the Upper East region.

## 5 Conclusion

In conclusion, our study underscores the clinical and epidemiological significance of *H. pylori* infection in northern Ghana, suggesting that fecal–oral transmission through street foods and raw produce is a public health concern. The high prevalence of *H. pylori* observed, coupled with significant associations with environmental and behavioral factors, underscores the need for targeted public health interventions. Efforts should prioritize improving sanitation, waste management, food hygiene, and access to clean water to reduce the transmission of *H. pylori* and its associated health risks.

## Author Contributions

**Elvis Ramsy Awuni**: conceptualization, study design, data acquisition, and drafting of manuscript. **Maxwell Akonde**: performed the data analysis, interpretation, and revising the manuscript. **Benedict Boateng Antuamwine**: conceptualization, supervision, data analysis, interpretation, and writing of the manuscript. All authors reviewed and approved the final version for publication.

## Funding

The authors have nothing to report.

## Ethics Statement

Ethical approval for the study was obtained from the Quality Assurance and Ethics Committee of the University for Development Studies, Tamale (SAHS‐QAERC‐MLS/600‐02/0015/2018‐2019).

## Consent

Administrative permission was obtained from each facility, whereas informed consent for the study and scientific publications was obtained from each patient. For respondents below 18 years, their parents or legal guardians consented on their behalf.

## Conflicts of Interest

The authors declare no conflicts of interest.

## Data Availability

The data that support the findings of this study are available from the corresponding author upon request.
